# Root Responses to Boron Deficiency Mediated by Ethylene

**DOI:** 10.3389/fpls.2015.01103

**Published:** 2016-01-08

**Authors:** Agustín González-Fontes, M. B. Herrera-Rodríguez, Esperanza M. Martín-Rejano, M. T. Navarro-Gochicoa, Jesús Rexach, Juan J. Camacho-Cristóbal

**Affiliations:** Departamento de Fisiología, Anatomía y Biología Celular, Universidad Pablo de OlavideSevilla, Spain

**Keywords:** abscisic acid, auxin, boron deficiency, calcium signaling, ethylene, primary root, reactive oxygen species, root hairs

## Abstract

Low boron (B) supply alters the architecture of the root system in *Arabidopsis thaliana* seedlings, leading to a reduction in the primary root growth and an increase in the length and number of root hairs. At short-term (hours), B deficiency causes a decrease in the cell elongation of the primary root, resulting in a lower growth. Experimental approaches using ethylene insensitive *Arabidopsis* mutants, inhibitors of ethylene response, and GUS reporter lines suggest that ethylene is involved in these responses of the primary root to B deficiency. Furthermore, it has been shown that auxin participates in the inhibition of cell elongation under short-term B deprivation. These results support that an interaction between ethylene and auxin plays an important role in controlling the primary root elongation, in which a number of genes related to the synthesis, transport, and signaling of both phytohormones could modulate this effect. Evidence for a root cross-talk among both hormones and other possible intermediates (abscisic acid, calcium sensors, and reactive oxygen species) in response to B deficiency is provided and discussed.

## Role of Boron in Plant Development

Boron (B)—an element with properties intermediate between metals and non-metals–is an essential nutrient for vascular plants, and its limited availability affects yields and quality of crops producing significant economic losses (Blevins and Lukaszewski, 1998; Tanaka and Fujiwara, 2008).

Three mechanisms to explain B uptake and transport in plants have been described: (i) passive diffusion through the plasma membrane, (ii) facilitated diffusion through channels (NIPs, nodulin 26-like intrinsic proteins), and (iii) active high-affinity transport mediated by BOR transporters and induced under low B availability (Brown et al., 2002; Takano et al., 2008; Miwa and Fujiwara, 2010; Reid, 2014).

Both boric acid and borate have the ability to react with compounds containing *cis*-diol groups resulting in stable borate ester complexes. Thus, the best-known role of B is its structural function in the cell wall, where borate acts to form a diester bond between apiose residues of two rhamnogalacturonan II monomers; this dimer—the first molecule linked by borate identified in the plant kingdom—contributes to the steadiness of the cell wall (Ishii and Matsunaga, 1996; Kobayashi et al., 1996; O’Neill et al., 1996). In addition, B has been related to other two main processes, namely, the maintenance of plasma membrane integrity through the formation of complexes with compounds containing *cis*-diol moieties (e.g., glycoproteins and glycolipids) and the support of metabolic activities, so that its deficiency affects numerous metabolic and physiological processes that take place during both reproductive and vegetative stages of a plant’s life cycle (Blevins and Lukaszewski, 1998; Brown et al., 2002; Bolaños et al., 2004; Goldbach and Wimmer, 2007; Camacho-Cristóbal et al., 2008a). To explain this apparently pleiotropic effect of B, it has been proposed that the main role of B is the stabilization of *cis*-hydroxyl-containing molecules, irrespectively of their function (Bolaños et al., 2004). Nonetheless, with the exception of the primary structural role of B in the cell wall, so far there is not a hypothesis which fully explains how so many plant processes are affected by short-term B deficiency.

## Ethylene-Auxin Interaction in the Control of Root Development

Plant hormones regulate many aspects of growth and differentiation in plants, often through interaction between them. Thus, without exception, auxin, cytokinin, and ethylene are involved in regulation of root development (Ruzicka et al., 2007, 2009).

The roles of auxin and ethylene in controlling plant development have been thoroughly studied. It is well known that these two hormones act synergistically in regulating certain developmental processes, such as formation and elongation of root hairs, but also that they act antagonistically in other processes such as the development of lateral roots and hypocotyl elongation (Stepanova et al., 2007). The cross-talk between both hormones can be analyzed from the signaling pathways of ethylene and auxin (Muday et al., 2012). A first interaction occurs by activation of genes containing promoter regions that respond to ethylene and auxin, allowing both signaling pathways to directly regulate transcription. A second interaction occurs through the expression of genes that are auxin responsive, but whose activities regulate the synthesis, signaling or the response of ethylene, and vice versa (Muday et al., 2012). Therefore, ethylene and auxin can interact at three levels: reciprocally regulating their biosynthesis, influencing the response pathway, or acting on the same genes (Stepanova et al., 2007).

In some processes of plant growth and differentiation, auxin and ethylene can cause similar responses due to the capacity of auxin to promote ethylene synthesis by increasing ACC (1-aminocyclopropane-1-carboxylic acid) synthase activity. Exogenous application of IAA results in increased transcription of multiple genes responsible for ACC synthase (ACS), leading to an increase in ethylene production (Liang et al., 1992; Stepanova et al., 2007; Benková and Hejátko, 2009). Nevertheless, it has also been described that ethylene modulates the synthesis, transport, and auxin signaling in processes such as root growth and the formation of root hairs (Benková and Hejátko, 2009; Muday et al., 2012). Thus, while auxin can inhibit root growth in the absence of ethylene, ethylene inhibits root growth by increased auxin levels in certain areas of the root (Stepanova et al., 2007). For instance, ethylene enhances shootward auxin transport from the root apical to elongation zone by upregulating the transcription of AUX1 and PIN2, which mediate auxin delivery into cells of the elongation zone. Increased auxin levels elicit auxin responses in this zone that decrease cell elongation (Ruzicka et al., 2007).

Therefore, the maintenance of an appropriate ethylene-auxin balance is one of the most important mechanisms involved in root growth regulated by both hormones (Benková and Hejátko, 2009).

## Boron Availability Affects the Formation of Root Hairs and the Root System Growth *Via* Ethylene

In vascular plants, the most rapid response to B deficiency is the growth inhibition of both primary and lateral roots (Dell and Huang, 1997). Furthermore, this mineral deficiency elicits root hair formation and elongation (Takano et al., 2006; Martín-Rejano et al., 2011) and a decrease in the cell elongation of the primary root (Dell and Huang, 1997; Camacho-Cristóbal et al., 2015). These changes in root architecture can seriously affect the ability of plants to take up water and nutrients.

### Number and Length of Root Hairs

**FIGURE 1:**
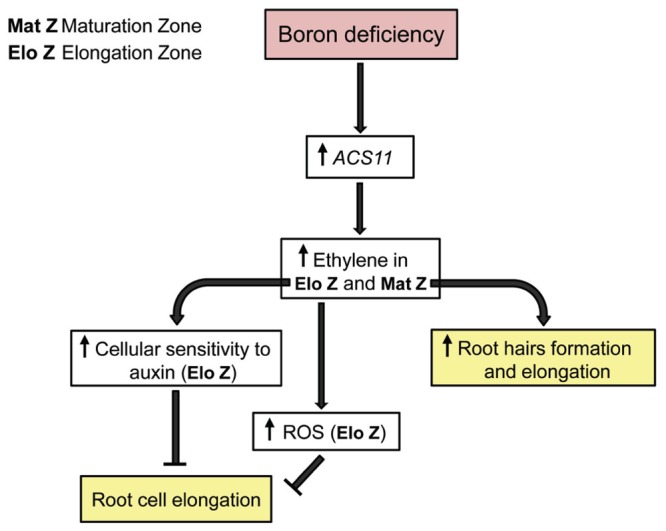
**Proposed model for short-term response of the primary root of *Arabidopsis thaliana* plants to B deficiency**. For more details see the text.

Interestingly, low B supply (0.4 μM) leads to an increase in the length and number of root hairs even after only 1 day of B deficiency (Martín-Rejano et al., 2011). This effect appears to be mediated by ethylene (**Figure 1**), which is supported by the following three facts: first, both the ethylene reporter EBS::GUS and the ACS11::GUS lines showed an increased expression in the maturation zone of primary root in response to low B supply (Martín-Rejano et al., 2011) and B deficiency (Camacho-Cristóbal et al., 2015), respectively, which suggests an accumulation of ethylene in this root zone; second, B limiting-induction of root hairs disappeared in an ethylene insensitive *(ein2-1) Arabidopsis thaliana* mutant (Martín-Rejano et al., 2011), which shows that the ethylene signal transduction to the nucleus through EIN2 protein is required to induce the formation and elongation of root hairs under conditions of limiting B; third, the effect of low B supply (0.4 μM, Martín-Rejano et al., 2013) or B deficiency (Camacho-Cristóbal et al., 2015) on root hair length was attenuated in the presence of Ag^+^—an inhibitor of ethylene response. In agreement with these results, ethylene has been reported to induce the formation and elongation of root hairs in *Arabidopsis* (Grierson et al., 2014 and references therein).

Additionally, it is well known that reactive oxygen species (ROS) are necessary for root hair growth in *Arabidopsis* (Foreman et al., 2003). In fact, *Arabidopsis rhd2* mutants, which lack respiratory burst oxidase homolog C (RBOHC, a plasma membrane NADPH oxidase), have markedly decreased levels of ROS and, consequently, form shorter root hairs; treatment of *rhd2* roots with ROS partly suppressed the mutant phenotype (Foreman et al., 2003). However, it has been reported that *Arabidopsis rhd2* plants grown on B-free solidified media formed root hairs, which were similar to those of control plants (Bassil et al., 2005). B lack in *Arabidopsis rhd2* mutants could induce the formation of root hairs by two non-exclusive hypotheses: (i) another mechanism in which a notable participation of ROS would not be essential, and/or (ii) the increase in the activity of other RBOs expressed in *Arabidopsis* roots. Consistent with this last hypothesis, NADPH oxidase activities increased in primary roots of *Arabidopsis* after short-term B deficiency (Camacho-Cristóbal et al., 2015).

Finally, it is noteworthy to mention that the higher number and length of root hairs under B limitation could be an efficient way to enhance the B uptake by NIP5;1 proteins localized to the plasma membrane of root hairs (Takano et al., 2006).

### Primary Root Growth

Low B treatment (0.4 μM) for 1 to 4 days alters the architecture of the root system in *Arabidopsis* seedlings leading to a reduction in the primary root growth (Martín-Rejano et al., 2011). It is well known that ethylene plays critical roles in modulating root growth (Le et al., 2001; Swarup et al., 2007). The signals generated by ethylene converge in transcription factors, such as EIN3, which trigger a transcriptional cascade resulting in activation and repression of hundreds of genes (Stepanova and Alonso, 2009). Interestingly, EBS::GUS activity also increased in the elongation zone of the *Arabidopsis* primary roots treated with limiting B (Martín-Rejano et al., 2011). This suggests that the accumulation of ethylene mediates the inhibition of primary root growth under low B supply. In addition to ethylene, auxin seems to be involved in the inhibition of *Arabidopsis* primary root treated with limiting B supply (0.4 μ M). Two facts support this assumption: (i) the expression of the auxin reporter DR5::GUS increased in the elongation zone of primary roots and (ii) the growth of primary roots in the auxin resistant *aux1-22* mutant was less sensitive to low B treatment than in wild-type plants (Martín-Rejano et al., 2011).

Total primary root growth depends on two developmental processes: the division of cells in the meristematic region and the elongation of cells that leave the root meristem (Scheres et al., 2002). Several abiotic stresses such as B toxicity cause a decrease in meristem size because of a progressive reduction of cell division, which correlates with the inhibition of root growth (Aquea et al., 2012). However, other abiotic stresses, including B deficiency, affect mainly cell elongation in the growing tissues of plants (Dell and Huang, 1997; Martín-Rejano et al., 2011; Camacho-Cristóbal et al., 2015). Thus, a short-term B deficiency has been shown to strongly inhibit the elongation of root cells as manifested by their short length, which results in a rapid inhibition of primary root growth (Camacho-Cristóbal et al., 2015). This inhibition in cell elongation can reasonably be attributed to the adverse effects of B deprivation on the physical stability of the cell wall, which is essential for the cell elongation process (De Cnodder et al., 2005). In fact, changes in cell wall polysaccharides and the structural proteins can moderate plant cell expansion during development (Cosgrove, 1997). Cell area is increased in an order of magnitude along the root elongation phase, and this requires a major restructuring of the cell wall and an increase in polysaccharide biosynthesis (Tsang et al., 2011). Hence, growing primary roots are sensitive to the cell wall damage. For instance, inhibition of cellulose biosynthesis or interference in the cell wall assembly rapidly reduced elongation (Tsang et al., 2011). Therefore, the structural damages in the cell wall caused by B deficiency, together with the downregulation of several cell wall-related genes (Camacho-Cristóbal et al., 2008b), could lead to disorders that affect cell elongation. Even though the processes that control the extent of root cell elongation under B deficiency are still not clearly defined, there is growing evidence supporting the mediation of ethylene (Camacho-Cristóbal et al., 2015). Thus, interestingly, *ACS11* gene, which encodes an isoform of ACC synthase, was rapidly overexpressed in the absence of B, a fact consistent with the increased expression of ACS11::GUS reporter line in the root elongation zone (Martín-Rejano et al., 2014; Camacho-Cristóbal et al., 2015). It has been shown that the expression of *ACS* genes increases when there are severe developmental problems (Tsuchisaka et al., 2009). Although *ACS11* gene expression was rapidly induced under B deficiency, this is not the case with other genes of the *ACS* family (Camacho-Cristóbal et al., 2015). These results agree with those obtained by Tsang et al. (2011) in which only the *ACS11* gene was induced in the root elongation zone upon treatment with the cell wall-damaging isoxaben. This rapid upregulation of the *ACS11* gene under B deficiency would suggest an enhancement of ACC and/or ethylene synthesis in *Arabidopsis* roots to inhibit expansion of cells leaving the root meristem (Le et al., 2001; Swarup et al., 2007). In addition, cell elongation of the ethylene-insensitive mutant *ein2-1* was less sensitive to B deficiency than that of the wild-type plants (Camacho-Cristóbal et al., 2015), which also supports the occurrence of this ethylene-dependent pathway to control the inhibition of cell elongation under B deficiency.

Ethylene, or ACC, causes an irreversible blockage in cell elongation from which cells cannot expand (Le et al., 2001; De Cnodder et al., 2005). Inhibition of root elongation is an evident effect of ethylene or ACC, which is a synergistic effect with auxin on this process (Rahman et al., 2001; Swarup et al., 2002). Analysis of inhibition of root growth by ethylene and auxin revealed that both reduce the rate of cell expansion in the central zone of elongation (Rahman et al., 2007; Swarup et al., 2007). Importantly, IAA exogenous application to *Arabidopsis* seedlings grown under control conditions decreased root cell elongation in a similar way to that caused by B deprivation (Martín-Rejano et al., 2014). This would support that B deficiency induces a decrease in root cell elongation, in part, by increased levels of auxin. In addition, the higher expression of the auxin reporter IAA2::GUS in the elongation zone and the complete restoration of cell elongation by PEO-IAA—an auxin signaling inhibitor—in B-deficient roots indicate the requirement of auxin signaling in the response of cell elongation to B deficiency (Martín-Rejano et al., 2014; Camacho-Cristóbal et al., 2015). It has also been suggested that the shootward auxin transport *via* AUX1 and PIN2 proteins participates in this response, since the primary root growth in *aux1-22* and *pin2* mutants was less sensitive to B deprivation than in wild-type plants (Martín-Rejano et al., 2013; Camacho-Cristóbal et al., 2015).

Furthermore, an accumulation of ROS has been reported in the elongation zone of *Arabidopsis* roots when they were subjected to B deprivation, and that these early responses to B deficiency were mediated by ethylene probably acting upstream of ROS production (Oiwa et al., 2013; Camacho-Cristóbal et al., 2015). Interestingly, it has also been described that localized auxin accumulation increases ROS levels (Peer et al., 2013), which would also explain the observed accumulation of ROS in the elongation zone under B deficiency. In this regard, the shorter cell elongation in *Arabidopsis* roots under B deficiency has been related to oxidative damage by ROS (Camacho-Cristóbal et al., 2015), which can be produced by NADPH oxidases of plasma membrane (Suzuki et al., 2011). In fact, it has been proposed that the crosslinking between hydroxyproline-rich glycoproteins driven by ROS could be an important mechanism to inhibit root cell elongation (De Cnodder et al., 2005). Consistent with this, NADPH oxidase activity in *Arabidopsis* roots was significantly higher after short-term B deprivation and, in addition, diphenyleneiodonium, which inhibits ROS generation by NADPH oxidases, attenuated the effect of B deficiency on cell elongation, even in the presence of ACC (Camacho-Cristóbal et al., 2015). These results support the hypothesis of a relation between ethylene, auxin, ROS production and inhibition of root cell elongation under this mineral deficiency.

## Role of Ethylene and Auxin in Root Response to the Boron Deficiency: Which Works First?

B deficiency causes a decrease in root growth that is mediated by ethylene and auxin, but which of the two phytohormones acts first triggering this response? When *Arabidopsis* seedlings grown with 10 μM B were treated with ACC, their primary root cell lengths decreased up to a similar size to those grown under B deficiency suggesting the involvement of ethylene in the B deficiency-induced response (Camacho-Cristóbal et al., 2015). Interestingly, when auxin signaling was inhibited by PEO-IAA in B-deficient seedlings, the length of their root cells increased up to the size of those treated with B sufficiency, even in the presence of ACC (Martín-Rejano et al., 2013, 2014; Camacho-Cristóbal et al., 2015). Furthermore, the blockage of ethylene signaling by Ag^+^ was able to abolish the effect of B deprivation on IAA2::GUS expression (Camacho-Cristóbal et al., 2015). These findings suggest that auxin signaling acts downstream of the ethylene signal in the root response to B deficiency, that is, ethylene would be acting previously to auxin. Consistent with these results, it has been described that the effect of ethylene on the root growth is largely mediated by an increase in the auxin response, which results in a lower elongation of root cells (Swarup et al., 2007).

According to this, a potential model is proposed to explain how seedling roots of *A. thaliana* respond in short-term to B deprivation, and how ethylene and auxin are associated with this response (**Figure 1**). B deficiency would trigger an increase in *ACS11* gene expression and, consequently, in the levels of ACC and ethylene. This rise would lead to alter auxin response in the primary root of *Arabidopsis* plants that, in turn, result in a decrease of the root cell length (Swarup et al., 2007; Muday et al., 2012). The increased auxin response in the elongation zone could explain the results observed with the auxin reporter line IAA2::GUS under B deficiency (Camacho-Cristóbal et al., 2015).

An intriguing fact is why B deficiency provokes a decrease in the cell elongation of the primary root while increases length of root hairs. It is well known that Ca^2+^ and ROS are necessary for root hair growth in *Arabidopsis* (Foreman et al., 2003; Takeda et al., 2008; Monshausen et al., 2009; Swanson et al., 2011). As B deprivation increases cytosolic levels of Ca^2+^ (Quiles-Pando et al., 2013; González-Fontes et al., 2014) and ROS (Oiwa et al., 2013; Camacho-Cristóbal et al., 2015) in the *Arabidopsis* roots, the higher levels of both could explain the enhanced length of root hairs under this mineral deficiency. However, under B deficiency, the auxin level in the elongation zone would exceed the threshold value of growth inhibition inducing local responses that inhibit the cell elongation.

Finally, and interestingly, it has recently been reported that abscisic acid (ABA) signaling activates two Ca^2+^-dependent protein kinases—CPK4 and CPK11—that are capable of phosphorylating ACSs resulting in increased ethylene production, which inhibits primary root growth in *Arabidopsis* (Luo et al., 2014); as discussed by these authors, ABA would be acting not only upstream of ethylene, but also affecting auxin accumulation and/or auxin signaling *via* ROS production (Luo et al., 2014, and references therein). Moreover, it was shown that also a Ca^2+^-dependent protein kinase is necessary for the phosphorylation of RBOHD protein associated with ROS generation (Dubiella et al., 2013). Curiously, it has been reported that B deprivation increases cytosolic Ca^2+^ concentration in both tobacco BY-2 cells (Koshiba et al., 2010) and *Arabidopsis* roots (Quiles-Pando et al., 2013; González-Fontes et al., 2014), and that two encoding genes of Ca^2+^-dependent protein kinases *(CPK28* and *CPK29)* are also upregulated during short-term B deficiency (Quiles-Pando et al., 2013; González-Fontes et al., 2014). As B deprivation leads to increased levels of ethylene and ROS (Camacho-Cristóbal et al., 2015), in light of all these data it would not be ruled out that there might be a cross-talk among ABA, Ca^2+^ signaling, ethylene, auxin, and ROS in responses to different plant stresses, including B deficiency.

## Conflict of Interest Statement

The authors declare that the research was conducted in the absence of any commercial or financial relationships that could be construed as a potential conflict of interest.

## References

[B1] Aquea F. Federici F. Moscoso C. Vega A. Jullian P. Haseloff J. (2012). A molecular framework for the inhibition of *Arabidopsis* root growth in response to boron toxicity. *Plant Cell Environ.* 35 719–734. 10.1111/j.1365-3040.2011.02446.x 21988710

[B2] Bassil E. Nijo G. Baluska F. Volkmann D. Menzel D. Goldbach H. (2005). Boron deficiency rescues the *Arabidopsis thaliana* rhd2 root hair phenotype: are reactive oxygen species involved? *Eur. J. Cell Biol.* 84 63 10.1016/j.ejcb.2005.02.001

[B3] Benková E. Hejátko J. (2009). Hormone interactions at the root apical meristem. *Plant Mol. Biol.* 69 383–396. 10.1007/s11103-008-9393-6 18807199

[B4] Blevins D. G. Lukaszewski K. M. (1998). Boron in plant structure and function. *Annu. Rev. Plant Physiol. Plant Mol. Biol.* 49 481–500. 10.1146/annurev.arplant.49.1.481 15012243

[B5] Bolaños L. Lukaszewski K. Bonilla I. Blevins D. (2004). Why boron? *Plant Physiol. Biochem.* 42 907–912. 10.1016/j.plaphy.2004.11.002 15694285

[B6] Brown P. H. Bellaloui N. Wimmer M. A. Bassil E. S. Ruiz J. Hu H. (2002). Boron in plant biology. *Plant Biol.* 4 205–223. 10.1055/s-2002-25740

[B7] Camacho-Cristóbal J. J. Martín-Rejano E. M. Herrera-Rodríguez M. B. Navarro-Gochicoa M. T. Rexach J. González-Fontes A. (2015). Boron deficiency inhibits root cell elongation *via* an ethylene/auxin/ROS-dependent pathway in *Arabidopsis* seedlings. *J. Exp. Bot.* 66 3831–3840. 10.1093/jxb/erv186 25922480 PMC4473985

[B8] Camacho-Cristóbal J. J. Rexach J. González-Fontes A. (2008a). Boron in plants: deficiency and toxicity. *J. Integr. Plant Biol.* 50 1247–1255. 10.1111/j.1744-7909.2008.00742.x 19017112

[B9] Camacho-Cristóbal J. J. Herrera-Rodríguez M. B. Beato V. M. Rexach J. Navarro-Gochicoa M. T. Maldonado J. M. (2008b). The expression of several cell wall-related genes in *Arabidopsis* roots is down-regulated under boron deficiency. *Environ. Exp. Bot.* 63 351–358. 10.1016/j.envexpbot.2007.12.004

[B10] Cosgrove D. J. (1997). Relaxation in a high-stress environment: the molecular bases of extensible cell walls and cell enlargement. *Plant Cell* 9 1031–1041. 10.1105/tpc.9.7.1031 9254929 PMC156977

[B11] De Cnodder T. Vissenberg K. Van Der Straeten D. Verbelen J.-P. (2005). Regulation of cell length in the *Arabidopsis thaliana* root by the ethylene precursor 1-aminocyclopropane-1-carboxylic acid: a matter of apoplastic reactions. *New Phytol.* 168 541–550. 10.1111/j.1469-8137.2005.01540.x 16313637

[B12] Dell B. Huang L. (1997). Physiological response of plants to low boron. *Plant Soil* 193 103–120. 10.1023/a:1004264009230

[B13] Dubiella U. Seybold H. Durian G. Komander E. Lassig R. Witte C. P. (2013). Calcium-dependent protein kinase/NADPH oxidase activation circuit is required for rapid defense signal propagation. *Proc. Natl. Acad. Sci. U.S.A.* 110 8744–8749. 10.1073/pnas.1221294110 23650383 PMC3666735

[B14] Foreman J. Demidchik V. Bothwell J. H. F. Mylona P. Miedema H. Torres M. A. (2003). Reactive oxygen species produced by NADPH oxidase regulate plant cell growth. *Nature* 422 442–446. 10.1038/nature01485 12660786

[B15] Goldbach H. E. Wimmer M. (2007). Boron in plants and animals: is there a role beyond cell-wall structure? *J. Plant Nutr. Soil Sci.* 170 39–48. 10.1002/jpln.200625161

[B16] González-Fontes A. Navarro-Gochicoa M. T. Camacho-Cristóbal J. J. Herrera-Rodríguez M. B. Quiles-Pando C. Rexach J. (2014). Is Ca^2+^ involved in the signal transduction pathway of boron deficiency? New hypotheses for sensing boron deprivation. *Plant Sci.* 217–218, 135–139. 10.1016/j.plantsci.2013.12.011 24467905

[B17] Grierson C. Nielsen E. Ketelaar T. Schiefelbein J. (2014). Root hairs. *Arabidopsis Book* 12 e0172 10.1199/tab.0172 PMC4075452 24982600

[B18] Ishii T. Matsunaga T. (1996). Isolation and characterization of a boron-rhamnogalacturonan-II complex from cell walls of sugar beet pulp. *Carbohydr. Res.* 284 1–9. 10.1016/0008-6215(96)00010-9

[B19] Kobayashi M. Matoh T. Azuma J. (1996). Two chains of rhamnogalacturonan II are cross-linked by borate-diol ester bonds in higher plant cell walls. *Plant Physiol.* 110 1017–1020. 10.1104/pp.110.3.1017 12226238 PMC157802

[B20] Koshiba T. Kobayashi M. Ishihara A. Matoh T. (2010). Boron nutrition of cultured tobacco BY-2 cells. VI. Calcium is involved in early responses to boron deprivation. *Plant Cell Physiol.* 51 323–327. 10.1093/pcp/pcp179 20008940

[B21] Le J. Vandenbussche F. Van Der Straeten D. Verbelen J.-P. (2001). In the early response of *Arabidopsis* roots to ethylene, cell elongation is up- and down-regulated and uncoupled from differentiation. *Plant Physiol.* 125 519–522. 10.1104/pp.125.2.519 11161008 PMC1539361

[B22] Liang X. Abel S. Keller J. A. Shen N. F. Theologis A. (1992). The 1-aminocyclopropane-1-carboxylate synthase gene family of *Arabidopsis thaliana. Proc. Natl. Acad. Sci. U.S.A.* 89 11046–11050. 10.1073/pnas.89.22.11046 PMC50480 1438312

[B23] Luo X. Chen Z. Gao J. Gong Z. (2014). Abscisic acid inhibits root growth in *Arabidopsis* through ethylene biosynthesis. *Plant J.* 79 44–55. 10.1111/tpj.12534 24738778

[B24] Martín-Rejano E. M. Camacho-Cristóbal J. J. Herrera-Rodríguez M. B. Navarro-Gochicoa M. T. Rexach J. González-Fontes A. (2014). Boron deprivation inhibits root cell elongation *via* an ethylene/auxin/ROS-dependent pathway. International Symposium on Plant Signaling and Behavior. 2014 Delhi, Abstract 46 Available at: http://ds9.botanik.uni-bonn.de/zellbio/AG-Baluska-Volkmann/pnb2014/program_oral.pdf

[B25] Martín-Rejano E. M. Camacho-Cristóbal J. J. Herrera-Rodríguez M. B. Rexach J. Navarro-Gochicoa M. T. González-Fontes A. (2011). Auxin and ethylene are involved in the responses of root system architecture to low boron supply in *Arabidopsis* seedlings. *Physiol. Plant.* 142 170–178. 10.1111/j.1399-3054.2011.01459.x 21338369

[B26] Martín-Rejano E. M. Camacho-Cristóbal J. J. Herrera-Rodríguez M. B. Rexach J. Navarro-Gochicoa M. T. González-Fontes A. (2013). The inhibition of root cell elongation under boron deficiency is mediated by ethylene and auxin. XVII International Plant Nutrition Colloquium. Istanbul, Abstract 640–641. ISBN: 978-605-4348-62-6.

[B27] Miwa K. Fujiwara T. (2010). Boron transport in plants: co-ordinated regulation of transporters. *Ann. Bot.* 105 1103–1108. 10.1093/aob/mcq044 20228086 PMC2887066

[B28] Monshausen G. B. Bibikova T. N. Weisenseel M. H. Gilroy S. (2009). Ca^2+^ regulates reactive oxygen species production and pH during mechanosensing in *Arabidopsis* roots. *Plant Cell* 21 2341–2356. 10.1105/tpc.109.068395 19654264 PMC2751959

[B29] Muday G. K. Rahman A. Binder B. M. (2012). Auxin and ethylene: collaborators or competitors? *Trends Plant Sci.* 17 181–195. 10.1016/j.tplants.2012.02.001 22406007

[B30] Oiwa Y. Kitayama K. Kobayashi M. Matoh T. (2013). Boron deprivation immediately causes cell death in growing roots of *Arabidopsis thaliana* (L.) *Heynh. Soil Sci. Plant Nutr.* 59 621–627. 10.1080/00380768.2013.813382

[B31] O’Neill M. A. Warrenfeltz D. Kates K. Pellerin P. Doco T. Darvill A. G. (1996). Rhamnogalacturonan-II, a pectic polysaccharide in the walls of growing plant cell, forms a dimer that is covalently cross-linked by a borate ester. *J. Biol. Chem.* 271 22923–22930. 10.1074/jbc.271.37.22923 8798473

[B32] Peer W. A. Cheng Y. Murphy A. S. (2013). Evidence of oxidative attenuation of auxin signalling. *J. Exp. Bot.* 64 2629–2639. 10.1093/jxb/ert152 23709674

[B33] Quiles-Pando C. Rexach J. Navarro-Gochicoa M. T. Camacho-Cristóbal J. J. Herrera-Rodríguez M. B. González-Fontes A. (2013). Boron deficiency increases the levels of cytosolic Ca^2+^ and expression of Ca^2+^-related genes in *Arabidopsis thaliana* roots. *Plant Physiol. Biochem.* 65 55–60. 10.1016/j.plaphy.2013.01.004 23416496

[B34] Rahman A. Amakawa T. Goto N. Tsurumi S. (2001). Auxin is a positive regulator for ethylene-mediated response in the growth of *Arabidopsis* roots. *Plant Cell Physiol.* 42 301–307. 10.1093/pcp/pce035 11266581

[B35] Rahman A. Bannigan A. Sulaman W. Pechter P. Blancaflor E. B. Baskin T. I. (2007). Auxin, actin and growth of the *Arabidopsis thaliana* primary root. *Plant J.* 50 514–528. 10.1111/j.1365-313X.2007.03068.x 17419848

[B36] Reid R. (2014). Understanding the boron transport network in plants. *Plant Soil* 385 1–13. 10.1007/s11104-014-2149-y

[B37] Ruzicka K. Ljung K. Vanneste S. Podhorská R. Beeckman T. Friml J. (2007). Ethylene regulates root growth through effects on auxin biosynthesis and transport-dependent auxin distribution. *Plant Cell* 19 2197–2212. 10.1105/tpc.107.052126 17630274 PMC1955700

[B38] Ruzicka K. Simásková M. Duclercq J. Petrásek J. Zazímalová E. Simon S. (2009). Cytokinin regulates root meristem activity *via* modulation of the polar auxin transport. *Proc. Natl. Acad. Sci. U.S.A.* 106 4284–4289. 10.1073/pnas.0900060106 19246387 PMC2657394

[B39] Scheres B. Benfey P. Dolan L. (2002). Root development. *Arabidopsis Book* 1:e0101 10.1199/tab.0101 PMC3243376 22303222

[B40] Stepanova A. N. Alonso J. M. (2009). Ethylene signaling and response: where different regulatory modules meet. *Curr. Opin. Plant Biol.* 12 548–555. 10.1016/j.pbi.2009.07.009 19709924

[B41] Stepanova A. N. Yun J. Likhacheva A. V. Alonso J. M. (2007). Multilevel interactions between ethylene and auxin in *Arabidopsis* roots. *Plant Cell* 19 2169–2185. 10.1105/tpc.107.052068 17630276 PMC1955696

[B42] Suzuki N. Miller G. Morales J. Shulaev V. Torres M. A. Mittler R. (2011). Respiratory burst oxidases: the engines of ROS signaling. *Curr. Opin. Plant Biol.* 14 691–699. 10.1016/j.pbi.2011.07.014 21862390

[B43] Swanson S. J. Choi W.-G. Chanoca A. Gilroy S. (2011). *In vivo* imaging of Ca^2+^, pH, and reactive oxygen species using fluorescent probes in plants. *Annu. Rev. Plant Biol.* 62 273–297. 10.1146/annurev-arplant-042110-103832 21370977

[B44] Swarup R. Parry G. Graham N. Allen T. Bennett M. (2002). Auxin crosstalk: integration of signalling pathways to control plant development. *Plant Mol. Biol.* 49 411–426. 10.1023/A:1015250929138 12036264

[B45] Swarup R. Perry P. Hagenbeek D. Van Der Straeten D. Beemster G. T. S. Sandberg G. (2007). Ethylene upregulates auxin biosynthesis in *Arabidopsis* seedlings to enhance inhibition of root cell elongation. *Plant Cell* 19 2186–2196. 10.1105/tpc.107.052100 17630275 PMC1955695

[B46] Takano J. Miwa K. Fujiwara T. (2008). Boron transport mechanisms: collaboration of channels and transporters. *Trends Plant Sci.* 13 451–457. 10.1016/j.tplants.2008.05.007 18603465

[B47] Takano J. Wada M. Ludewig U. Schaaf G. Von Wirén N. Fujiwara T. (2006). The *Arabidopsis* major intrinsic protein NIP5; 1 is essential for efficient boron uptake and plant development under boron limitation. *Plant Cell* 18 1498–1509. 10.1105/tpc.106.041640 16679457 PMC1475503

[B48] Takeda S. Gapper C. Kaya H. Bell E. Kuchitsu K. Dolan L. (2008). Local positive feedback regulation determines cell shape in root hair cells. *Science* 319 1241–1244. 10.1126/science.1152505 18309082

[B49] Tanaka M. Fujiwara T. (2008). Physiological roles and transport mechanisms of boron: perspectives from plants. *Eur. J. Physiol.* 456 671–677. 10.1007/s00424-007-0370-8 17965876

[B50] Tsang D. L. Edmond C. Harrington J. L. Nühse T. S. (2011). Cell wall integrity controls root elongation *via* a general 1-aminocyclopropane-1-carboxylic acid-dependent, ethylene-independent pathway. *Plant Physiol.* 156 596–604. 10.1104/pp.111.175372 21508182 PMC3177261

[B51] Tsuchisaka A. Yu G. Jin H. Alonso J. M. Ecker J. R. Zhang X. (2009). A combinatorial interplay among the 1-aminocyclopropane-1-carboxylate isoforms regulates ethylene biosynthesis in *Arabidopsis thaliana. Genetics* 183 979–1003. 10.1534/genetics.109.107102 PMC2778992 19752216

